# Re-evaluating the choice of gamma stimulation frequency for the potential treatment of Alzheimer’s disease: Novel invisible spectral flicker evokes gamma responses at various frequencies

**DOI:** 10.1371/journal.pone.0321633

**Published:** 2025-05-20

**Authors:** Mark Alexander Henney, Bianca Laura Hansen, Luna Skytte Hansen, Manja Gersholm Grønberg, Martin William Thorning-Schmidt, Henrik Enggaard Hansen, Mai Nguyen, Paul Michael Petersen, Line Katrine Harder Clemmensen, Marcus Carstensen, Kristoffer Hougaard Madsen

**Affiliations:** 1 Technical University of Denmark, Department of Applied Mathematics and Computer Science, Kgs. Lyngby 2800, Denmark; 2 OptoCeutics ApS, Copenhagen 2200, Denmark; 3 Technical University of Denmark, Department of Physics, Kgs. Lyngby 2800, Denmark; 4 Technical University of Denmark, Department of Electrical and Photonics Engineering, Kgs. Lyngby 2800, Denmark; 5 Copenhagen University Hospital Amager and Hvidovre, Danish Research Centre for Magnetic Resonance; Centre for Functional and Diagnostic Imaging and Research, Hvidovre 2650, Denmark; Museo Storico della Fisica e Centro Studi e Ricerche Enrico Fermi, ITALY

## Abstract

With recent advances in the potential usage of visual gamma stimulation at 40 Hz for the treatment of Alzheimer’s disease, there is motivation to evaluate adjacent frequencies to ensure that specifically 40 Hz is optimal. As visual stimulation with luminance flicker may affect adherence in clinical trials due to its inherent perceived flickering, invisible spectral flicker (ISF) was proposed as a more comfortable alternative for entraining 40 Hz. Based on current understanding of the potential mechanism of action for 40 Hz stimulation, the exact frequency is debatable. This study investigates the ability of ISF to evoke acute gamma responses at several frequencies in the range of 36–44 Hz. Twenty healthy volunteers were included in an electroencephalography (EEG) experiment with ISF stimulation at nine different frequencies (36–44 Hz, 1 Hz interval). Estimated signal-to-noise ratios (SNR) showed that the cortical power was significantly increased at all stimulation frequencies compared to baseline, but with no significant difference in SNR between stimulation frequencies. There was a significant subject effect, suggesting that there is higher variability between subjects than between frequencies alone. Our results indicate that ISF can evoke steady-state potentials at several frequencies in the low gamma range of 36–44 Hz. Across the population of participants, no preference or trend for any specific gamma stimulation frequency in the tested range was found. While the subject-stimulus interaction was significant, it described little variance and showed no specific patterns for individual preference of frequency.

## Introduction

### Alzheimer’s disease and current state-of-the-art

By 2050, it is anticipated that more than 132 million individuals will be affected by dementia globally [1]. The associated annual costs are projected to reach 2.8 trillion dollars in 2050 compared to 1.3 trillion in 2019 [2]. While several disorders can lead to dementia, the most common cause of dementia is Alzheimer’s disease (AD) [3]. Alzheimer’s dementia manifests with cognitive decline which progresses over time at the detriment of patients and relatives.

Recently, there has been increasing success in the development of disease-modifying pharmacological therapies with monoclonal antibodies that target the removal of Amyloid-beta (Aβ). However, the effects observed are limited to about 30% reduction in disease progression, and the treatment is associated with some inconvenience and potential risk of brain oedema and brain haemorrhage from the Aβ-clearance process [4].

Concurrently, there is growing interest in the interpretation of AD as a dysfunction of the brain’s neural networks, where the electrophysiological abnormalities are investigated as pathological markers and prospective treatment targets (this topic is vast and has been covered extensively by others, including but not limited to [5–8]). As a results of this, investigations have emerged into the potential for using 40 Hz brain stimulation based on visual flicker for the purpose of AD therapy [9–19]. However, little is understood about the mechanism of action (MoA) for how 40 Hz stimulation could slow progression of AD, and part of this understanding begs the question: *Why exactly 40 Hz*? In some cases, 40 Hz stimulation is compared to (relatively) low- and high-frequency stimulation [9] as a kind of placebo control. However, such comparisons to frequencies outside the mechanistically relevant vicinity of 40 Hz would not reveal nuances about the specificity of 40 Hz.

This study investigates the ability of a principally novel light technology, *invisible spectral flicker* [20] (ISF), to evoke a cortical response at other frequencies in the vicinity of 40 Hz. Invisible spectral flicker is distinct from luminance flicker (LF) used in [9–13, 15, 16], in which the brightness is modulated in time, and from heterochromatic flicker (HF), in which the light alternates between two colours. By definition, ISF generalises to alternating between two different but arbitrarily complex spectra of light. If the two spectra are composed to be perceived as sufficiently similar, it may reduce the sensation of flicker or render it entirely invisible under some circumstances (see e.g. [21] for a review of flicker fusion). We have previously demonstrated that the implementation of ISF investigated in [17, 18] and in the ongoing clinical trial ClinicalTrials.gov ID NCT05260177 is associated with significantly lower perception of flicker and self-reported discomfort compared to LF and red/green HF [22]. Selecting the appropriate spectra for stimulation is not trivial, but we have previously investigated which parts of the visual spectrum are most important to represent [23]. From that study, comparing the evoked cortical responses to all unique HF combinations of six different colours, we concluded that it is more important to include either extreme of the visual spectrum (i.e. blue or red) in at least one of the flicker phases, than it is for the two phases to be very spectrally distinct.

While flickering visual stimuli at frequencies in the 40 Hz range have have been demonstrated previously [24, 25], we aim to widen the basic understanding of cortical responses to ISF at various frequencies. As detailed below, two observations may favour modification of the stimulation frequency: (1) hypotheses for the mechanism of action (MoA) are not tied to exactly 40 Hz, and (2) the perception of flicker is frequency dependent.

### Motivation for changing stimulus frequency

There are multiple hypotheses for the MoA of 40 Hz stimulation AD therapy. The work conducted by Cardin *et al*. [26] shows a key role of fast-spiking (FS) parvalbumin-positive (PV+) inhibitory interneurons in generating gamma oscillations in the cortical network with notable sensitivity to 35-55 Hz modulated optogenetic activation. This contrasts with the peak sensitivity of pyramidal neurons at approximately 8 Hz. Additionally, targeting PV+ and somatostatin-positive (SST+) interneurons responsible for gamma oscillations seems to be the right approach, as there is a cell-specific dependency on the propagation of visual flicker [27]. This is aligned with [28], highlighting a hippocampal circuit resonating at 39 ± 3 Hz (mean ± sd) when activated with a choline agonist. Furthermore, PV+ and SST+ interneurons have been linked to neurological disorders like AD and could be a causal step in the disease pathogenesis [29]. However, none of these systems expose a necessity for stimulation to be at *exactly* 40 Hz. On the contrary, they may provide evidence of a wider dynamic range of applicable frequencies.

According to the IEEE Recommended Practices for Modulating Current in High-Brightness LEDs for Mitigating Health Risks to Viewers, the risk of biological (side) effects such as seizures, headaches, or malaise is highest from exposure to visible flicker [30]. Gamma stimulation applications that make use of the sensory pathway(s) may be especially affected by the stimulation frequency. This includes 40 Hz visual flicker as used in [13–19], in which the amount of perceived flicker is affected by the flicker frequency [21]. As such, modifying the stimulation frequency could enhance the utility and treatment adherence of such therapies, though the clinical implications are speculative.

Under the speculative assumption that small deviations from 40 Hz can be tolerated without loss of potential clinical benefit, it is worth reconsidering the frequency. If the sensitivity distribution for input frequencies to the FS-PV+ interneurons presented in [26] were to guide the choice of stimulation frequency, it would suggest increasing the stimulation frequency. This is important as the perception of flicker is reduced at higher frequencies which can be exploited to improve the user experience. Contrarily, the resonance frequency distribution of hippocampal circuits presented in [28] would point to a slight reduction in the preferred frequency. This would have the opposite implications for the amount of perceived flicker.

Previous studies have shown the ability to visually evoke steady-state potentials (SSVEPs) with invisibly flickering stimuli [24, 31–33]. The point at which flicker becomes *invisible*—the critical flicker fusion frequency (CFF)—has been studied extensively for decades. It is known to depend on many parameters including but not limited to the perceiver, ambient lighting, and light modulation depth, duty cycle, luminance, and varies across a wide frequency band depending on circumstances[30, 31, 34, 35]. Reconsidering stimulation frequency may be especially valuable for *invisible spectral flicker* (ISF) [20] (as used in [17, 18]) which aims to reduce the amount of perceivable flicker to the point of being “merely perceptible” by alternating between two complex spectra of light, not limited to monophasic spectra of single-colours (see Fig. 2). The results of [22] would indicate that 40 Hz is close to the CFF of ISF for some observers (participants) under the reported circumstances (though this was not explicitly tested) and that increasing the frequency could make the flicker even less perceptible or *im*perceptible. Thus, it is relevant to verify that ISF can evoke SSVEPs above 40 Hz and evaluate whether any frequency preference is evident from the evoked power.

**Fig 1:**
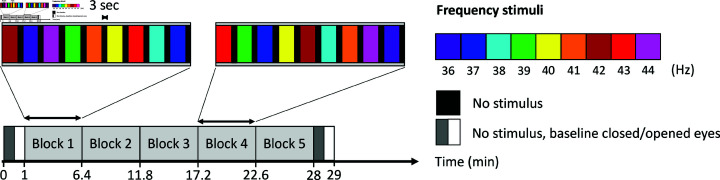
Overview of experimental design: Nine ISF stimulation conditions with frequencies varying from 36–44 Hz in 1 Hz increments were repeated five times in a randomised complete block design (RCBD). Within each block, the nine frequency conditions were shown once. The stimulation trials had a duration of 30 seconds and were separated by a three second inter-trial interval. Before and after the RCBD, a 1-minute resting-state baseline was recorded—30 seconds with eyes closed and 30 seconds with eyes open.

**Fig 2:**
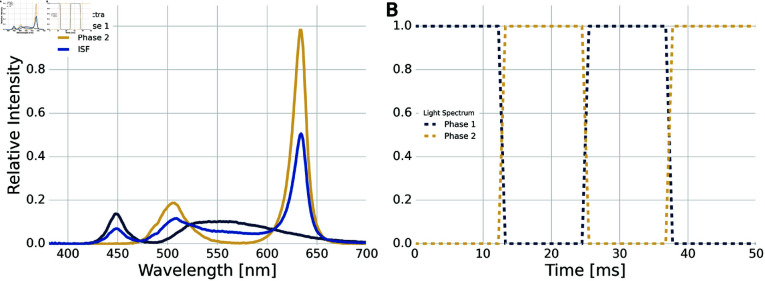
Invisible spectral flicker light specification: Invisible spectral flicker alternates at 40 Hz between two phases of spectrally different white lights which ease the fusion and reduce the perception of flicker. **A:** Spectral profiles of individual phases and the composite which defines ISF. One phase is a colder white, while the other is a warmer white, but both span broad bands of the visible spectrum. The combined spectrum is the average of the two phases achieved by 50% duty cycle between them. **B:** Schematic of 40 Hz temporal colour mixing with square waveforms and 100% modulation depth to achieve ISF.

In this study, we investigate the ability of ISF to evoke cortical responses at several stimulation frequencies in the range of 36–44 Hz. This is proximal to and symmetrically distributed around the current choice of 40 Hz used in [17, 18] (and the ongoing study NCT05260177), overlaps with the interneuron sensitivity- and hippocampal resonance frequency distributions from [26, 28], and is identical to the clinically relevant band used by [36–38] for differentiating AD patients from geriatric controls. The study design and objectives are similar to those of [25], but the visual stimulation paradigms are qualitatively different. They investigate the [32–50] Hz band (in two-hertz increments) using visual stimulation with single colour luminance flicker of white, red, green, or blue, thus making it an excellent study for comparison.

## Materials and methods

### Experimental design

Twenty healthy volunteers (10 males, 10 females) with an age between 21 to 64 years (an average of 30 years) were included in an electroencephalography (EEG) experiment (approved by the institutional review board at DTU Compute under the protocol number: COMP-IRB-2021-01) between May 28th and July 24th of 2021. Participants were recruited conveniently by word-of-mouth and were not reimbursed for participating. The data was collected in accordance with the Declaration of Helsinki, and all participants gave written informed consent prior to data collection. All subjects had either normal or corrected to normal vision, and the exclusion criteria included history of migraine, photosensitive epilepsy, or other known neurological disorders. Subjects were situated in front of a visual stimulation device (Light Therapy System (LTS), OptoCeutics ApS, Denmark) at a distance of 60 cm and were requested to sit calmly, minimise unnecessary movement and to look at the device while the stimulation light was on. The EEG recording was conducted with a 19 channel dry electrode system (Zeto Inc., CA, USA) with a sampling rate of 500 Hz and mounted according to the 10–20 system, with linked mastoid reference. Recording was done using cloud-based software (the Zeto Cloud Platform), a 24-bit analogue-to-digital converter (ADC), and no additional live filtering after the ADC.

The EEG consisted of an initial baseline recording of one minute, of which the first 30 seconds was with eyes closed (EC) and the latter 30 seconds with eyes open (EO). Following this, a stimulation paradigm ensued, during which subjects were exposed to ISF at nine integer frequencies from 36–44 Hz with no concomitant task. The experiment followed a randomised complete block design as sketched in Fig. 1, where the order of stimulus frequencies was randomised within each of the five blocks, resulting in a total of 45 stimulation trials per subject. Trials were separated by a three second inter-trial-interval without stimulation (light off) to avoid leakage between them. The duration between blocks was identical to the inter-trial-interval, tying the five blocks into one continuous run. When including the baseline EO and EC trials, the total number of trials per subject was 49, resulting in a grand total of 980 trials. The total duration of the experiment was about 29 minutes.

With exception of the varying temporal modulation frequency, the light specifications of the ISF stimulation was similar to the parameters presented in [20] and used in [17, 18] with a luminance of $1800\;{cd/m}^{2}$. The waveform was controlled with pulse-width modulation (PWM) operating at 2.93 kHz and a modulation depth of 100%. The PWM dimming values were defined individually for each of the coloured LEDs in two different sets to achieve the two colour profiles needed to fuse the alternating phases of ISF light. The individual and combined spectra of the the two phases of ISF and the 40 Hz temporal mixing are shown in Fig.2. While the term *invisible spectral flicker* was coined in [20] to reflect a subtype of chromatic flicker with reduced flicker fusion frequency, no tests were conducted in to estimate the participants’ perceptibility of the flicker as a function of frequency.

### EEG processing

Processing of the EEG was done using custom scripts written in Python (version 3.8) using the open-source MEG+EEG analysis package MNE-Python (version 1.3.0). All recordings were manually inspected and marked for periods with high-amplitude noise. All trials containing excessively noisy segments were removed, reducing the total number of trials from 980 to 597. This high number of rejections resulted in slight imbalance of the factors and non-completion among the subject-stimulus combinations. As the noise typically was persistent over a couple of consecutive trials, sub-segmenting and rejection of shorter durations of bad data did not improve the fraction of rejected data.

Each recording was filtered with a finite impulse response (FIR) Hamming notch filter at 50 Hz with 0.5 Hz band width and 0.5 Hz transition bandwidth. Subsequently, they were highpass filtered with an FIR Hamming filter with lower cutoff of 1 Hz and transition bandwidth of 1 Hz. No lowpass filtering took place. Finally, the channels were re-referenced to a common average.

From the pre-processed EEG data, the power spectral density (PSD) of each channel *m* in trial *n* was estimated by a 30 s periodogram given by (Eq 1):

$${PSD}_{n,m}(f)=\frac{1}{L}{\left|\sum_{l=1}^{L}x_{n,m}e^{-j2\pi fl}\right|}^{2},\;f=-F,-F+\frac{f_{s}}{L},\ldots ,F,$$ (1)

in which *L* is the number of samples in the trial, *j* is the unit imaginary number, *f*_*s*_ = 500 Hz is the sampling frequency, and $F=\frac{f_{s}}{2}=250$ Hz is the Nyquist frequency. The PSDs of the occipital channels (O1, O2) were averaged according to (Eq 2) to obtain the mean occipital PSD for trial *n*:

$${PSD}_{n,O}(f)=\frac{1}{2}\left({PSD}_{n,O1}(f)+{PSD}_{n,O2}(f)\right).$$ (2)

**Fig 3:**
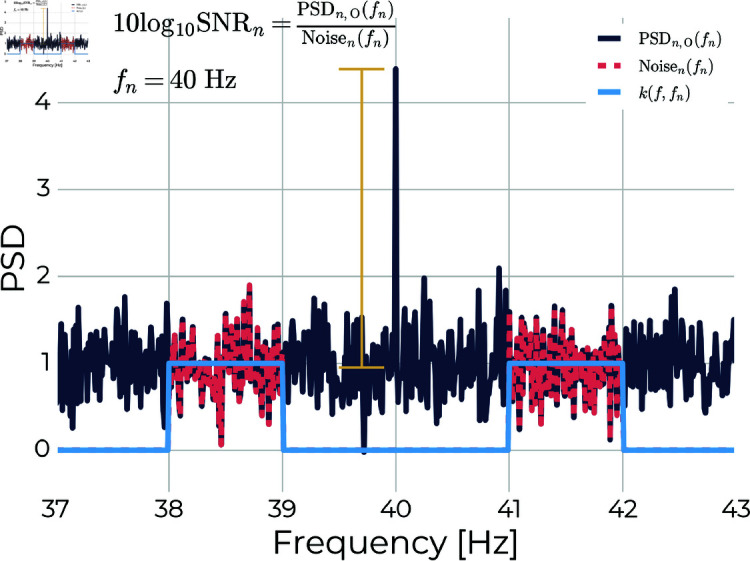
Estimating signal-to-noise ratio. The SNR of a given trial, *n*, was estimated as the occipital power at the stimulation frequency, *f*_*n*_, normalised to the noise defined as the average power in the neighbouring bands (see Eqs 1 to (5).

The signal-to-noise ratio (SNR) was calculated as the power at the stimulus frequency, *f*_*n*_, for trial *n* normalised to the average power in the neighbouring bands (frequency bins within ±2 Hz, but excluding the nearest ±1 Hz) according to (Eq 3) and sketched in Fig. 3:

$${SNR}_{n}=10log_{10}\left(\frac{{PSD}_{n,O}(f_{n})}{{Noise}_{n}(f_{n})}\right),$$ (3)

where the denominator noise term given by (Eq 4) estimates the mean power on either side of *f*_*n*_ within the trial:

$${Noise}_{n}(f_{n})=\frac{\sum_{f}{PSD}_{i,O}(f)⊙k(f,f_{n})}{\sum_{f}k(f,f_{n})},$$ (4)

where ⊙ is the Hadamard product, and *k*(*f*,*f*_*n*_) is the kernel that selects the noise neighbourhood according to (Eq 5):

$$k(f,f_{n})=\left\{ \begin{array}{cc} 1 & \text{ for }f-2\;Hz<f_{n}<f-1\;Hz\text{ and }f+1\;Hz<f_{n}<f+2\;Hz\\ 0 & \text{ Otherwise } \end{array}\right..$$ (5)

For the baseline condition, in which no stimulation took place, the SNR was calculated at 40 Hz under the assumption that it according to Eq 3 would be 0 (or at least equivalent) for any frequency relative to its neighbours. This assumption is fair as the PSD in the baseline conditions at rest is primarily aperiodic and thus follows approximately a 1/*f*^*a*^ shape.

### Statistial analysis

The relationship between ISF stimulation frequency (stimulus) and occipital response SNR was investigated using a linear mixed-effects model. The initial model used SNR as the dependent variable and three main factors as independent variables: stimulus, subject, and block, as well as all their two-way interactions. The stimulus frequency was considered a fixed factor, while the subjects and blocks were modelled as random effects with variable intercepts.

The initial linear mixed effects model of the experiment was described by (Eq 6), in which all terms were present prior to model reduction. Here, ${\mathrm{SNR}}_{ijk}$ refers to the SNR response to the trial with stimulus *i*, subject *j*, and block *k*:

$${\mathrm{SNR}}_{ijk}=\mu +\alpha_{i}+a_{j}+b_{k}+c_{jk}+d_{ik}+f_{ij}+\varepsilon_{ijk},\;$$ (6)

where


i=0,1,…,9,j=1,2,…,20,k=0,1,…,5,

$$a_{j}\sim 𝒩\left(0,\sigma_{a}^{2}\right),$$

$$b_{k}\sim 𝒩\left(0,\sigma_{b}^{2}\right),$$

$$c_{jk}\sim 𝒩\left(0,\sigma_{c}^{2}\right),$$

$$d_{ik}\sim 𝒩\left(0,\sigma_{d}^{2}\right),$$

$$f_{ij}\sim 𝒩\left(0,\sigma_{f}^{2}\right),$$

$$\varepsilon_{ijk}\sim 𝒩\left(0,\sigma^{2}\right),$$

and α, *a*, and *b* are the stimulus, subject, and block main effects, respectively, while *c*, *d*, and *f* are the subject-block, stimulus-block, and stimulus-subject interaction effects, respectively, and $𝒩\left(\mu ,\sigma^{2}\right)$ denotes a Gaussian distribution with mean μ and variance $\sigma^{2}$. Note that the 0th index for stimulus and block correspond to the baseline condition.

We implemented the initial model defined by (Eq 6) in the statistical computing software R (version 4.3.0) using the ‘lme4‘ package (version 1.1-33) [39] and fit the model to the data using a restricted maximum likelihood. The initial model fit was checked for variance homogeneity, normality of residuals and random effects, independence of residuals, and uncaptured residual structure. Finally, the influence of single observations was checked by means of the Cook’s distance. One observation was found to be particularly influential (Cook's distance>1.25). However, through re-analysis removing this observation from the data, it was confirmed that the significant factors were unchanged (though the estimated mean SNR for the baseline condition was closer to the expected value of 0 in the re-analysis without this observation). Ultimately, the observation was included in the final analysis, as no reason was found to exclude it. One subject had SSVEP responses consistently an order of magnitude higher than the rest, so the influence of this subject on the model was tested. We fitted a corresponding model to the data without that subject to check its influence and verify that the significant terms did not change. As exclusion of the subject did not alter the significant terms, the subject was ultimately included in the analysis.

The random effects structure of our model was simplified by way of likelihood ratio tests which test the null hypothesis $H_{0}:\sigma_{r}^{2}=0$ against the alternative $H_{A}:\sigma_{r}^{2}>0$, where $\sigma_{r}^{2}$ was a given random effect variance. We adjusted the p-values by a factor 1/2 to account for testing on the boundary of the positive variance constraint. Non-significant random effects were eliminated in an iterative manner, where the effect with the highest p-value above α=5% was removed, and the reduced model was re-fitted. With this approach, the block main factor was found to be non-significant (p=0.5, see Table 1), but it was kept in the model, as it was both an integral part of the experimental design and involved in significant interactions. The stimulus-block interaction was not significant and was removed from the model.

**Table 1:** Initial model factor reduction.

Random Factor	LRT	DF	1/2 p-value
Subject	117.58	1	<0.001
Block	0.00	1	0.50
Block-Stimulus interaction	1.61	1	0.10
Subject-Block interaction	27.61	1	<0.001
Subject-Stimulus interaction	4.77	1	<0.001
**Fixed Factor**	**F-statistic**	**DF**	**p-value**
Stimulus	11.11	9	<0.001

Factors in the initial models are tested for significance to reduce model complexity using likelihood ratio tests (LRT) for random factors and maximum likelihood estimates for the fixed effect. Degress of freedom for a given factor is denoted DF. The Block factor was not significant, but as it was an integral part of the design and is implicit in significant interactions, it was decided to keep it in the model. The Block-Stimulus interaction was subsequently removed. As the LRT is a test on the boundary on the positive variance constraint, the p-values for random factors were corrected by a factor 1/2.

We proceeded to investigate the fixed effects structure by maximum likelihood estimation and found that the stimulus fixed effect was significant, thus deciding on Eq 7 as our final model to summarise the results:

$${\mathrm{SNR}}_{ijk}=\mu +\alpha_{i}+a_{j}+b_{k}+c_{jk}+f_{ij}+\varepsilon_{ijk},\;$$ (7)

where


i=0,1,…,9,j=1,2,…,20,k=0,1,…,5,

$$a_{j}\sim 𝒩\left(0,\sigma_{a}^{2}\right),$$

$$b_{k}\sim 𝒩\left(0,\sigma_{b}^{2}\right),$$

$$c_{jk}\sim 𝒩\left(0,\sigma_{c}^{2}\right),$$

$$f_{ij}\sim 𝒩\left(0,\sigma_{f}^{2}\right),$$

$$\varepsilon_{ijk}\sim 𝒩\left(0,\sigma^{2}\right),$$

α is the fixed stimulus effect, *a* and *b* are the random subject and block main effects, respectively, while *c* and *f* are the random subject-block and stimulus-subject interaction effects, respectively. The model assumptions were re-assessed for the final model by the same criteria as the initial model to verify that model reduction had not led to violation thereof.

In post-hoc analysis, the stimuli were compared in a pair-wise manner to each other and the baseline SNR estimates using paired t-tests with the Kenward-Roger degrees of freedom (DF) approximation. The p-values were adjusted by the Tukey method for a family of 45 hypotheses when comparing all stimuli, while they were corrected using Dunnett’s test when comparing them to the baseline.

## Results

**Fig 4:**
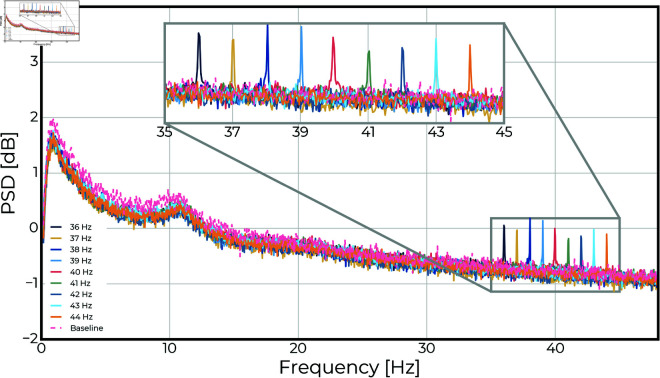
Grand averaged occipital EEG power spectra: The power spectral density estimates from each of the stimulation frequency conditions (and eyes-open baseline) are averaged across all repetitions and subject to obtain grand averages. As expected, the averaged power spectra show clear peaks at the stimulation frequencies. While there is some variability in the height of the peaks, it does not reveal a frequency of particularly increased resonance on the group level.

Fig. 4 presents the grand averaged estimates for the mean occipital EEG PSDs for each stimulus frequency (and baseline) conditions. From this, it is evident that, on average, all stimulus frequencies evoked occipital steady-state potentials at the corresponding frequencies, and that the average magnitude of the oscillatory power was comparable across the conditions, except for the baseline condition without stimulation.

**Fig 5:**
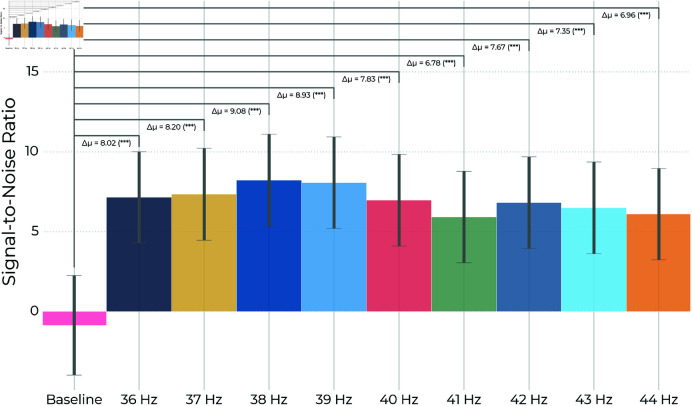
Estimated mean steady-state visually evoked potential: Estimated marginal means of the SSVEP responses and the baseline condition with no stimulation are plotted along with error-bars indicating their 95% confidence intervals (see also Table 2). Evidently, stimulation with all frequencies led to a significant increase in the SSVEP at the stimulation frequency compared to baseline (see also Table 3). Error bars indicate the 95% confidence intervals for the means.

Fig. 5 and Table 2 provide the estimated marginal means of the SNR and 95% confidence intervals based on Eq. 7 for each condition and the estimated contrast between each stimulus frequency and baseline. Evidently, all stimulation frequencies evoked an occipital response significantly higher than the baseline condition at their respective frequency. The highest response was observed from stimulating with 38 Hz, while the lowest was observed for 41 Hz. However, no particular pattern is noticeable, and none of the stimuli evoked significantly different responses compared to each other.

**Table 2:** Estimated marginal mean signal-to-noise ratios.

Stimulus	Est. Mean [dB]	SE	DF	Lower CL	Upper CL
Baseline	-0.86	1.53	30.82	-3.98	2.26
36 Hz	7.15	1.38	23.72	4.30	10.01
37 Hz	7.34	1.39	24.61	4.47	10.22
38 Hz	8.22	1.40	25.02	5.33	11.10
39 Hz	8.07	1.39	24.19	5.20	10.93
40 Hz	6.97	1.39	24.25	4.10	9.83
41 Hz	5.92	1.39	24.19	3.05	8.78
42 Hz	6.81	1.39	24.27	3.95	9.68
43 Hz	6.49	1.39	24.37	3.62	9.36
44 Hz	6.10	1.39	23.98	3.24	8.96

**Table 3:** Contrast of stimulus frequency vs. baseline.

Stimulus	Δ Baseline [dB]	SE	DF	t-statistic	p-value
36 Hz	8.02	0.98	23.72	8.15	<0.001
37 Hz	8.20	1.00	24.61	8.20	<0.001
38 Hz	9.08	1.01	25.02	8.98	<0.001
39 Hz	8.93	0.99	24.19	8.99	<0.001
40 Hz	7.83	1.00	24.25	7.87	<0.001
41 Hz	6.78	0.99	24.19	6.82	<0.001
42 Hz	7.67	0.99	24.27	7.71	<0.001
43 Hz	7.35	1.00	24.37	7.37	<0.001
44 Hz	6.96	0.97	23.98	7.06	<0.001

Estimated marginal means and corresponding 95% confidence intervals for the SNR of each stimulus factor level in Eq. 7. Standard-error of the mean is denoted SE, the degrees of freedom associated with the factor level is denoted DF, and the limits for the confidence intervals are denoted lower CL and upper CL, respectively.

**Fig 6:**
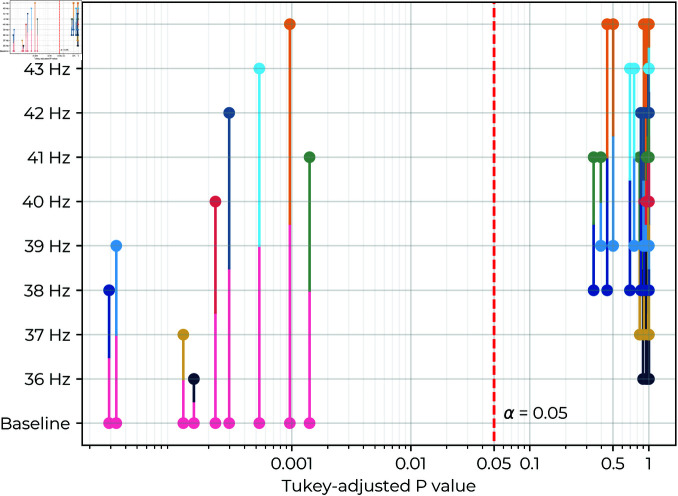
Pairwise p-values of stimulus contrasts: The p-values for estimated marginal mean differences in stimulus SNR are presented for each pair of stimuli. Dots map horizontally to their respective stimulus, and the positions of their connecting lines on the x-axis indicates the p-value for the comparison the stimuli. All comparisons of stimulation frequencies to the baseline conditions are clustered on the left (p<0.001), indicating significant difference. The comparisons between each pair of stimulation frequencies showed non-significant differences, as evident from their clustering to the right (p>0.05).

The EEG power (see Fig. 4) showed that stimulus SNR values were significantly higher than baseline for all frequencies (p<0.001, see Fig. 5 and Table 3), but no stimuli resulted in significantly different SNR (see Fig. 6). There was a significant subject effect (estimated variance: ${\hat{\sigma }}_{a}^{2}=32.83$; p<.001), and one subject responded 1-2 orders of magnitude higher than the others.

**Fig 7:**
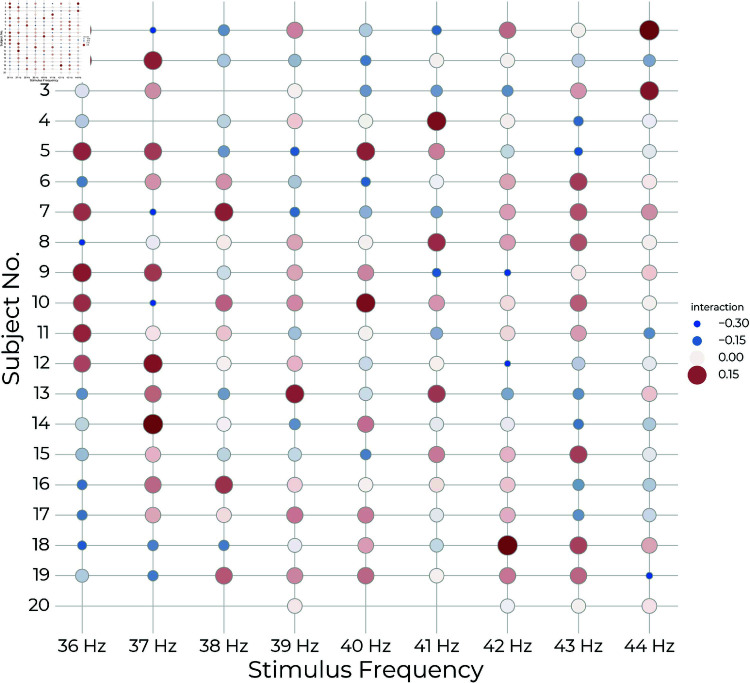
Subject-Stimulus random effect predictions: The random effect prediction for each subject and stimulus combination represents the individual subjects’ frequency preference, in which positive coefficient values indicate stronger individual response to a given frequency. Red and large circles indicate positive coefficients, blue and small circles indicate negative coefficients, and white and medium sized circles indicate coefficients close to zero. While the interaction effect was significant, the signs and sizes of the coefficients vary sporadically within subjects with no obvious patterns that would suggest individual preference for a sub-band of the tested range. Note that the high number of trial rejections resulted in slight imbalance of the factors and non-completion among the subject-stimulus combinations as is particularly evident for subject 20.

While the subject-stimulus interaction was significant, the explained variance is low (estimated variance: ${\hat{\sigma }}_{f}^{2}=1.1$; 1/2 p  = 0.019). Fig. 7 shows the random effect predictions for each level of the interaction term (combinations of subject and stimulus frequency). Notably, within each subject, the random effect prediction varies sporadically rather than tapering off around a maximum.

Estimated mean difference in SNR of the SSVEP at each stimulation frequency from baseline. All stimulation frequencies led to a significantly increase relative power compared to baseline. No pairs of stimulation frequencies resulted in significantly different increases in SNR. Standard-error of the mean difference is denoted SE, the degrees of freedom associated with the contrast is denoted DF. A slight imbalance of stimulation frequency representation across trials after data cleaning is evident from the variation in DFs.

Our results indicate that ISF can evoke SSVEPs at several frequencies in the low gamma range of 36-44 Hz. Across the population of participants, we found no preference or trend for any specific gamma stimulation frequency in the tested range.

## Discussion

The practice of using brain stimulation as a form of AD therapy is based on four decades of increasing knowledge about the relationship between AD and “40 Hz" physiological changes. However, the historical fixation with specifically 40 Hz has impacted state-of-the-art brain stimulation solutions that have now completed phase III clinical trials investigating the benefits for AD [19]. This is despite relying on mechanistic evidence that suggests that a broader band of stimulation frequencies may be feasible [26, 28].

While this study does not investigate the clinical efficacy of varying the stimulation frequency, it demonstrates that a wide range of stimulation frequencies in the vicinity of 40 Hz can evoke a strong and similar cortical response to ISF. We found no preference specifically for 40 Hz and no other obvious pattern of responses. On the contrary, the lack of frequency specificity may suggest that the other options are also feasible alternatives. This result is similar to that of [25] in their experiment comparing stimulation frequencies using white, red, green, or blue single colour luminance flicker. They found no significant differences in EEG SSVEP power over frequencies in the 32-50 Hz band. However, in another experiment where they compare the same stimulation frequencies across different light intensities ($\text{10-700}cd/m^{2}$), they found some frequencies to be significantly different [25]. In both experiments they observed a trend of power tapering off towards higher frequencies as was previously shown by [24]. A resonant effect when stimulating with 39 Hz was reported in [24], though this is neither observed in [25] nor our data.

Collectively, these studies demonstrate that the finer details of gamma stimulation optimisation may be both difficult to establish—even in the healthy population—and intrinsically dependent on the light configurations, evident perhaps by the many factors identified by IEEE on biological effects of visual flicker [30].

In addition to a strong subject effect, our results also showed a significant interaction effect between subject and stimulus frequency, though non-completion of the combinations limits the interpretation. An interaction effect could argue in favour of attempting to tune the stimulation frequency to individuals. However, there was no obvious pattern within each subject that pointed towards a frequency sub-band preference. If this effect reflected a resonant property within the individual, the coefficient would more likely be monotonically decreasing around the resonant peak. Instead, this result is likely to indicate sporadic test-retest variability more than evidence of individual frequency preference.

Others have investigated visual stimulation at and in the vicinity of the *individual gamma frequency* [40]. They found that endogenous gamma oscillations and input gamma oscillations co-exist with no evidence of entrainment between the systems and even originate from different sources in the visual cortex. While their experiments were conducted in the range of 50-90 Hz, the results suggest that steady-state input signals from visual stimulation are not coupling with and driving internal oscillators, but rather that the measured signal is a result of the filtering process (modulation amplitude and time delay) as the input propagates from the eyes to the visual cortex. This conclusion further complicates the interpretation for how intrinsic gamma changes observed in AD and stimulus evoked potentials relate. It also discourages use of the intrinsic gamma peak as a target for individual frequency optimisation.

Any change to the stimulation paradigm of ongoing or future studies of gamma stimulation should be motivated by a prospect of improved treatment effectiveness. This can be achieved by increasing the clinical efficacy or by improving the tolerability of the therapy and thus increasing the adherence to the treatment.

If it can be determined that other neighbouring frequencies to 40 Hz maintain clinical benefits, it is most likely beneficial to increase the frequency for visual gamma stimulation to improve the user experience. It is well established that the perception of flicker is reduced with increasing frequency and that this may reduce the side effects commonly associated with visible flicker [30]. Such a benefit to the tolerability might be especially noticeable for ISF whose flicker at 40 Hz is aimed to be *merely perceptible* [20]. It would have improved the interpretability of our study if the participants’ perceptability of the ISF stimulus as a function of stimulation frequency had been investigated. This could have been achieved by self-reported measures from the participants or unbiased behavioural tasks that test flicker perceptability such as those used in [41] or [42].

Even though the foundation for choosing 40 Hz is unspecific, and arguments can be made for improving comfort and safety with increased stimulation frequency, it is a challenge to provide sufficient evidence for altering the stimulation frequency for therapy of AD. Part of this is due to the progress made in clinical trials already, and altering the stimulation paradigm could require starting trials over at stage I. In practice, implementing a series of parallel clinical pipelines to simultaneously evaluate several choices of stimulation frequency would be infeasible.

An alternative approach would be to identify a reliable surrogate measure of clinical efficacy. Several suggestions have been made throughout time for physiological biomarkers of AD based on functional neuroimaging, including reduced "40 Hz" (36-44 Hz) EEG activity [36–38] and reduced default mode network activity during resting state functional magnetic resonance imaging [43, 44]. If these could be employed as acute measures of therapy relevance, it would be possible to properly optimise the stimulation frequency. Even better, the frequency could be tuned individually to provide the best trade-off between user comfort and efficacy. However, those metrics are presented as cross-sectional differences between groups and may not be feasible for assessing acute changes within the subject during stimulation.

In this study, we liberally assume that the acutely evoked gamma potential measured by occipital EEG is a reasonable indicator for future clinical benefit for AD patients. However, some researchers correctly identify this issue and investigates the expected correlation [45]. They compare the differences in acute 40 Hz power from visual- and auditory stimulation in [9] and [10] and relate it to the pre-clinical benefit for the AD mouse model. Here, they summarise that the long-term amyloid clearance is stronger in the study that achieved the highest evoked 40 Hz power by use of auditory stimulation. They provide supporting human data to show that the acute 40 Hz EEG power from auditory stimulation is higher than visual stimulation. That conclusion, however, does not take into account that the estimated EEG power is very dependent on the placement of reference electrodes (especially across stimulation modalities). This problem highlights the difficulty of estimating the correlation of even the simplest marker to clinical results.

To expand upon current understanding, it is imperative that forthcoming investigations delve further into the realm of hippocampal frequency-dependent circuitry, as well as the specific sensitivity of PV+ and SST+ cells to visual flicker. It is recommended that research efforts extend beyond the current threshold of 40 Hz. Such endeavours are crucial in advancing comprehension of the intricate workings of these neuronal populations, though the translation to clinical efficacy remains unknown. These considerations are also relevant in the context of other modes of 40 Hz stimulation, such as tactile [46], auditory [47], repetitive transcranial magnetic stimulation [48], and transcranial alternating current stimulation [49].

### Limitations of the study

Importantly, this study investigated the acutely evoked power during stimulation, and not the clinical implications for people with AD. It is currently unknown to which degree (if any) the evoked power is associated with clinical efficacy. This uncertainty has been addressed previously [45] and will require retrospective comparisons of clinical trial outcomes.

This study was conducted in a healthy and conveniently sampled cohort from the Copenhagen area of Denmark. Thus, interpretation of the results is limited by the homogeneity of the sample. This is particularly important in the context of interventions for AD, as our sample is demographically dissimilar to that population.

Our results clearly indicate an ability to evoke potentials at all stimulation frequencies, but the study was not powered to discern differences in evoked power between frequencies.
